# N-acetyl-L-tryptophan attenuates hepatic ischemia-reperfusion injury via regulating TLR4/NLRP3 signaling pathway in rats

**DOI:** 10.7717/peerj.11909

**Published:** 2021-08-10

**Authors:** Yitong Pan, Shuna Yu, Jianxin Wang, Wanzhen Li, Huiting Li, Chen Bai, Yaxin Sheng, Ming Li, Chenchen Wang, Jiao Liu, Peitong Xie, Can Wang, Jiying Jiang, Jianguo Li

**Affiliations:** 1 Department of Anatomy, Weifang Medical University, Weifang, Shandong, China; 2 2018 Grade 2 Glasses, Anaesthesiology Specialty, Weifang Medical University, Weifang, Shandong, China

**Keywords:** Hepatic ischemia-reperfusion injury, Pyroptosis, L-NAT, NLRP3

## Abstract

The aim of this study was to investigate the changes of TLR4/NLRP3 signal during hepatic ischemia-reperfusion injury (HIRI) and to verify whether N-acetyl-L-tryptophan (L-NAT) protected hepatocytes by regulating the activation of TLR4/NLRP3 signal. We have established the rat HIRI model and H_2_O_2_-induced cell damage model to simulate ischemia-reperfusion injury and detect the corresponding indicators. Compared with the sham group, Suzuki score and the level of serum ALT increased after HIRI, accompanied by an increased expression of NLRP3, ASC, Caspase-1, IL-1β, TLR4, and NF-κB. While L-NAT pretreatment reversed the above-mentioned changes. Compared with the control group, cells in the H_2_O_2_ treated group became smaller in cell volume and round in shape with unclear boundaries. Similar to the phenotypes in vivo, H_2_O_2_ treatment also induced significant increase in expression of pyroptosis-related proteins (NLRP3, ASC, Caspase-1 and IL-1β) and inflammatory factors (TLR4 and NF-κB). While L-NAT pretreatment attenuated injuries caused by H_2_O_2_. In conclusion, the present findings demonstrate that L-NAT alleviates HIRI by regulating activation of NLRP3 inflammasome, which may be related to the TLR4/NF-κB signaling pathway.

## Introduction

HIRI is a frequent complication of liver transplantation, partial hepatectomy, hemorrhagic shock and severe infection, which is also known as a common cause of clinical liver dysfunction. It is currently believed that excessive inflammation is critical for the pathogenesis of HIRI (Zhang et al., 2017). However, the mechanism by which HIRI causes inflammation is unclear. Recent evidence indicates that ischemia-reperfusion injury was triggered by a sterile inflammatory response through the inflammasome, which is composed of NLRs, ASC and pro-Caspase-1 (Li et al., 2020b). Among those inflammasome, NLRP3 is the most widely studied. Previous reported evidences suggested that NLRP3 inflammasome was activated in HIRI and down-regulate its downstream molecule IL-1β, which in turn induce the secretion of multiple cytokines (Tan et al., 2019; Kamo et al., 2013; Furuichi et al., 2006; Asmussen et al., 2016). Moreover, the severity of liver injury, the level of ROS, and the number of apoptotic hepatocytes were significantly attenuated once the activation of NLRP3, ASC, and Caspase-1 were inhibited in HIRI models (Huang et al., 2013; Zhu et al., 2011). The above studies suggest that the NLRP3 inflammasome plays an important role in the occurrence and development of HIRI. However, the upstream player responsible for initiating these responses remains unknown.

Damage associated molecular patterns (DAMPs), released from damaged cells during the process of ischemia-reperfusion, could not only initiate the assembly and activation of NLRP3 inflammasomes, but also be recognized by the pattern recognition receptor TLR4, which would promote the expression of cytokines and chemokines such as NLRPs, Pro-IL-1β, Pro-IL-18, TNF-α, IL-6, etc (Lu et al., 2018). Firstly, TLR4 signaling could induce the formation of immature IL-1β precursors. Secondly, the activation of NLRP3 inflammasomes could mediate the conversion of immature IL-1β precursors into mature IL-1β (Sharma et al., 2015). In summary, NLRP3 inflammasome and TLR4 signal work together to regulate inflammation. Recently, some scholars have also confirmed the role of TLR4/NLRP3 signaling in cystitis, nephritis and inflammation in monocytes after cardiopulmonary resuscitation (Butler et al., 2018). Therefore, we speculated that NLRP3 inflammasome and TLR4/NF- κB signals cooperate with each other in HIRI. However, the role of TLR4/NLRP3 signaling in HIRI has not been reported yet.

Substance P (SP), the endogenous inflammatory mediator, participates in the neurogenic inflammatory response induced by ischemia-reperfusion injury in a variety of tissues and organs by binding to its specific neurokinin-1 receptor (NK-1R) (Wu et al., 2009). Knockout the NK-1R gene or pretreatment with NK-1R antagonists could reduce the degree of cerebral ischemia reperfusion injury (Kalogeris et al., 2016), colitis (Corrigan, Vink & Turner, 2016), arthritis (Chang et al., 2013), pancreatitis (Pintér et al., 2014), respiratory tract inflammation (Martinez & Philipp, 2016), Concanavalin A-induced immune syndrome hepatitis (Thornton & Vink, 2015) and many other neurogenic inflammations reaction (Sirianni et al., 2015). Butler DSC et al. confirmed that the expression of NK-1R and SP in bladder mucosal nerve cells and bladder epithelial cells could be activated by bacterial infection and the inflammation could be inhibited by SR140333. NK-1R antagonists can reduce inflammation by regulating TLR4/NLRP3 signaling (Guo et al., 2004; Azzolina, Bongiovanni & Lampiasi, 2003). At present, the role of SP/NK-1R system has been reported in various types of liver diseases. Bang R and his colleagues (Bang et al., 2003) confirmed that NK-1R was expressed in hepatocytes. Pretreatment exogenous SP could mediate hepatocytes apoptosis, which could be inhibited by application of NK-1R antagonist or NK-1R KO. Garnier et al. (2016) found that the expression of NK-1R in hepatitis B was elevated, and NK-1R can be used as a target for hepatitis B treatment. Previous studies in our laboratory have also confirmed that L-NAT could relieve the damage induced by cerebral ischemia, hypoxia and H_2_O_2_ in cerebral cortex neurons (Yu et al., 2013), hippocampal neurons (Jiang et al., 2011), striatal neurons (Zhai et al., 2013) and NSC-34 cell (Sirianni et al., 2015), etc. Recently, we also confirmed the protective effect of L-NAT on hepatocytes during HIRI (Yu et al., 2013; Jiang et al., 2011). However, it is currently unclear whether the hepatoprotective effect of L-NAT is related to TLR4/NLRP3 signaling. Considering the role of the TLR4/NLRP3 signal in neuroinflammatory response and the protective effect of L-NAT on HIRI, we hypothesized that L-NAT may alleviate HIRI by inhibiting TLR4/NLRP3 signal. Therefore, we explored whether the NK-1R antagonist L-NAT could reduce HIRI by regulating TLR4/NLRP3 signaling pathways and its possible mechanism, which could provide experimental evidence for protective effects of L-NAT on HIRI.

## Materials and Methods

### Chemicals

L-NAT was obtained from Sigma Aldrich (St. Louis, MO., USA). Rat ELISA kit was purchased from NeoBioscience (Shanghai, China). The cell counting kit-8 (CCK-8) came from 7sea-Biotech (Shanghai, China). Anti-ASC, NLRP3, IL-1β, TLR4 and NF-κB were purchased from Bioss Antibodies (Beijing, China). Anti-Caspase 1 antibody came from Santa Cruz Biotechnology (Shanghai, China). TRIzol reagent was obtained from Life Technologies (USA). RTPA lysis was purchased from SolarBio Life Sciences (Beijing, China).

### Animals

Animal experiments were performed according to the guidelines for the use of laboratory animals, and were approved by the Animal Ethics Committee of Weifang Medical University (NO. 2019SDL040). Male Sprague-Dawley (SD) rats (6–8 weeks old), with an average weight of 200 to 220 g, were purchased from Pengyue experimental animal center (Jinan, China). They were randomly separated into three groups: sham group, I/R group and I/R + L-NAT group (*n* = 6 in each group). The rats were allocated in a temperature and humidity-controlled room (22 °C, 40–60% humidity) with a 12 h light/dark cycle. Rats were allowed free access to water, while fasted 12 h before surgery. According to previous studies, rats in the I/R + L-NAT group administrated L-NAT at a dose of 10 mg/kg through intraperitoneal injection, while those in the sham and I/R groups were given the same volume of saline 30 min before surgery (Li et al., 2020a).

Rats were anesthetized by 1% pentobarbital sodium (40 mg/kg) right before the surgery. The HIRI model was constructed by clamping the hepatic artery, portal vein, and common bile duct with a microvascular clamp for 45 min, then removing the clamp to restore reperfusion (Wang et al., 2019). The liver color changed from red to dark purple indicated that the model was successfully built. Rats in the sham group were subjected to laparotomy only, but no forceps were placed, and the anesthesia time was the same. After the deep anesthesia, the rats were euthanized after 6 h of reperfusion. Liver tissues and serum were collected for following morphological and ELISA test.

### Cell culture and treatment

The rat hepatocyte BRL cell line was purchased from the Cell Bank of the Chinese Academy of Sciences (Shanghai, China). The cells were divided into three groups: control group, H_2_O_2_ group and H_2_O_2_ + L-NAT group. BRL cells were cultured in 37 °C incubator with 95% air / 5% CO_2_. Therefore, BRL cells were exposed to 200 µM H_2_O_2_ with or without L-NAT for 18 h to establish the cell model. And H_2_O_2_+ L-NAT group was pre-treated with 10 µM L-NAT for 2 h before adding H_2_O_2_ (Wang et al., 2019). The control group was not treated.

### Cell viability assay

Cell viability was determined by CCK-8 colorimetric kit (7sea-Biotech, Shanghai, China). Medium containing L-NAT and H_2_O_2_ was removed and cells were washed by PBS after pretreatment. Then 100 µL of CCK-8 (5 mg/mL) in DMEM was added and incubated for 2 h at 37 °C. According to the product instructions, the optical density (OD) was measured by a microplate reader at wavelength of 450 nm. The cell viability was calculated using the following formula:

\begin{eqnarray*}\text{Cell viability}(\text{%})=\text{the absorbance of experimental group/the absorbance of control group}\nonumber\\\displaystyle \hspace*{72.0pt}\times 100\text{%} \end{eqnarray*}

### Liver Morphological Observation and pathological tissue score

Liver tissue specimens were immobilized with 4% paraformaldehyde for 24 h and embedded in paraffin. Liver sections (4 µm) were stained with hematoxylin and eosin (HE) for the evaluation of tissue damage by optical microscope.

The morphology results were evaluated by a pathologist who was not aware of the experimental groups and analyzed according to Suzuki histological criteria of liver injury. Hepatic injury-associated parameters such as edema, necrosis and ballooning degeneration were used to calculate the severity of tissue damage by a standard named Suzuki score.

### Immunofluorescence

BRL cells were exposed to H_2_O_2_ with or without L-NAT, fixed in 4% paraformaldehyde for 15 min and permeabilized with 0.3% Triton X-100-PBS for 15 min. Blocking was done in 5% goat serum for 30 min at 37 °C to inactivate endogenous peroxidase. Subsequent, the cells were incubated with anti-ASC antibody (1:200; Bioss), anti-NLRP3 antibody (1:200; Bioss), anti-IL-1β antibody (1:200; Bioss), anti-Caspase-1 antibody (1:100; Santa), anti-TLR4 (1:500; Bioss), and anti-NF-κB (1:500; Bioss) at 4 °C overnight and then incubated with FITC-conjugated secondary antibodies (1:150) at 37 °C for 30 min. DAPI staining was performed and image were taken using confocal microscopy (Olympus FV500; Olympus, Tokyo, Japan). Liver tissue from the sham group, I/R group and I/R + L-NAT group were made frozen sections of 10 µm thickness. And then immunofluorescence staining was performed using the method described above.

### Protein extraction and Western blotting

For protein extraction, each of 100 mg liver tissues were homogenized on ice in 1 ml RIPA lysis buffer with protease inhibitor mix PMSF. Lysates were obtained by centrifugation at 15,000 rpm for 15 min at 4 °C. The protein concentration was quantified by BCA method. Subsequently, protein samples were subjected to 10% SDS–polyacrylamide gel electrophoresis (SDS-PAGE) and transferred to a PVDF membrane, and then incubated with the following antibodies: anti-ASC antibody (1:200; Bioss), anti-NLRP3 antibody (1:200; Bioss), anti-TLR4 (1:500; Bioss) and anti-NF-κB (1:500; Bioss). Finally, the bands were observed using chemiluminescence (ECL) system according to the manufacturer’s instructions. Western blotting bands were analyzed using optical density scanning and Image Lab software (Bio-Rad). GAPDH (1:1000; Proteintech) was used as the loading control.

### Quantitative real-time PCR (qRT-PCR)

According to the manufacturer’s protocol, TRIzol agentia (Life, USA) was used to extract total RNA from liver tissues. PrimeScriptTM RT reagent kit was used for generating complementary DNA (cDNA) from RNA template by reverse transcription. Primers used in qRT-PCR were listed in Table 1. PCR was performed using cDNA as a template and TaqDNA polymerase. In brief, reverse transcription was proceeded under the conditions: the cDNA was pre-denatured at 94 °C for 3 min, and annealed 60 °C for 45 s for 39 cycles (Wang et al., 2019). GAPDH standardized the total mRNA for each gene in these experiments and the relative level was calculated using the previously reported 2^−*DDCT*^ method.

**Table 1:** Nucleotide sequences of primers used for qRT-PCR.

TLR4	F:5′-TCGAGCCAGAATGAGGACTG-3′
	R:5′-GATGATGTTGGCAGCAATGG-3′
NF-kB	F:5′-CTGGTGCATTCTGACCTTGC-3′
	R:5′-GGTCCATCTCCTTGGTCTGC-3′
GAPDH	F:5′-TGATTCTACCCACGGCAAGTT-3′
	R:5′-TGATGGGTTTCCCATTGATGA-3′
ASC	F:5′-GGCACAGCCAGAACAGAACA-3′
	R:5′-GCACGAACTGCCTGGTACTG-3′
NLRP3	F:5′-TGCAGCCTCACCTCACACTC-3′
	R:5′-AACCTCACAGAGCGTCACCA-3′
IL-1β	F:5′-CCTCGTGCTGTCGGACCCATA-3′
	R:5′-CAGGCTTGTGCTCTGCTTGTGA-3′
Caspase-1	F:5′-CCTCGTGCTGTCGGACCCATA-3′
	R:5′-CAGGCTTGTGCTCTGCTTGTGA-3′

### ELISA analysis

Rat ELISA kits (NeoBioscience, Shanghai, China) were used to measure the levels of IL-1β in the culture medium and ALT levels in rat serum according to the manufacturer’s instructions. The plates were read at 450 nm wave length by a microplate reader (Thermo, Shanghai, China).

### Statistical analysis

Both *in vivo* and *in vitro* experiments were repeated at least three times. Measurement data are presented as mean ± SD and were analyzed using SPSS 18.0 software (SPSS Inc., Chicago, IL, USA). One-way analysis of variance was utilized for comparison between groups and LSD test for pairwise comparison. Furthermore, *p* < 0.05 were considered statistically significant.

## Results

### Effect of L-NAT on hepatocytes morphology after HIRI

**Figure 1:**
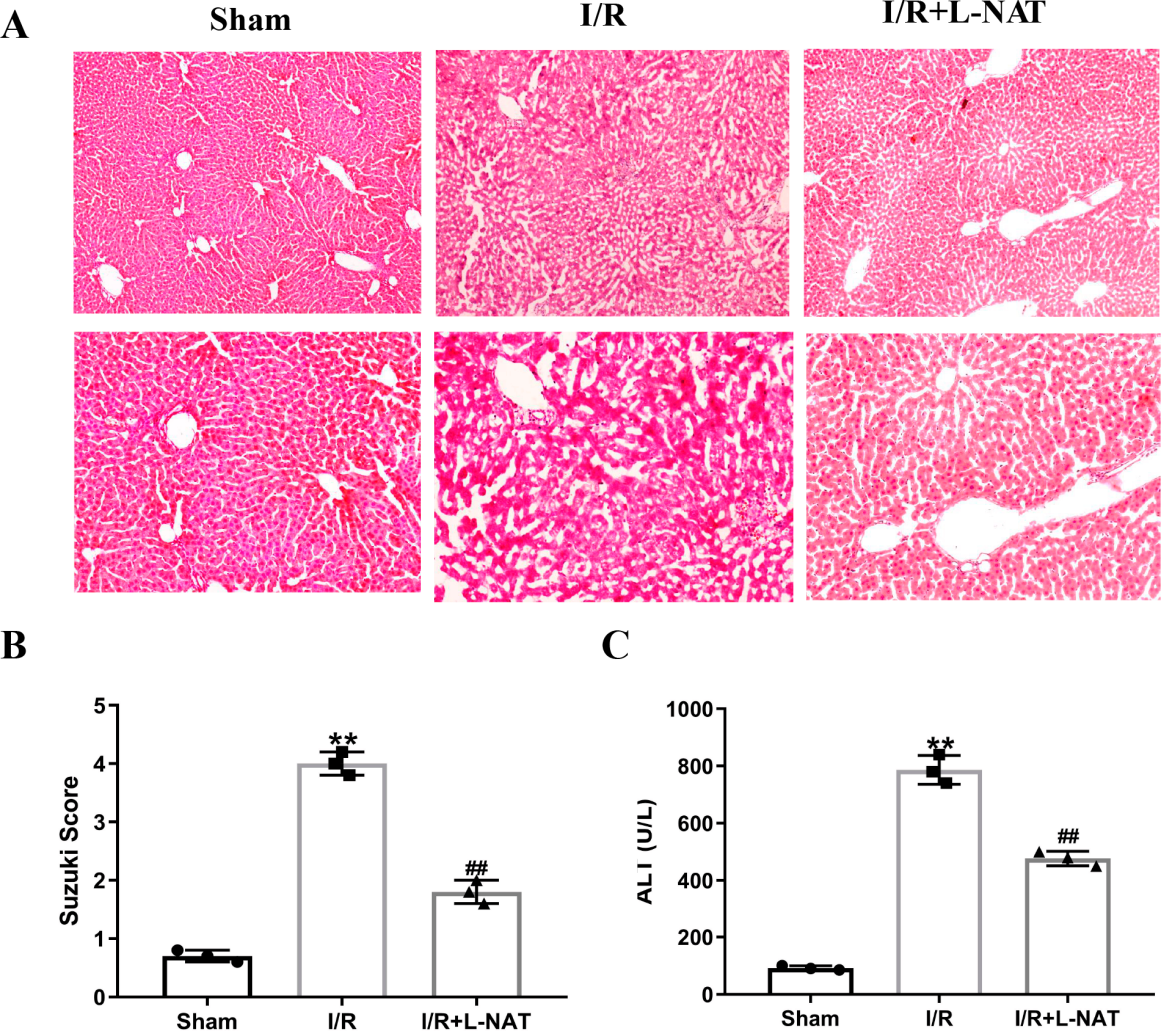
The rat hepatic ischemia-reperfusion injury (HIRI) model was verified. The HIRI model was constructed by clamping the hepatic artery, portal vein, and common bile duct with a microvascular clamp for 45 min, then removing the clamp to restore reperfusion. The L-NAT (10 mg/kg) or saline was administered by IP injection 30 min before ischemia, the rats of sham group were subjected to all surgical procedures except for I/R. The histological score was calculated according to the morphological changes of liver tissue (A). Compared with the sham group, the level of Suzuki score (B) and serum ALT (C) increased after HIRI. While L-NAT pretreatment reversed the changes. The data were the mean ± SD (One-way ANOVA, *n* = 3), ^∗∗^*p* < 0.01 compared with the sham group, ^##^*p* < 0.01 compared with the I/R group.

To evaluate the cytoprotective effect of L-NAT on morphological changes of hepatocytes, we constructed a rat 70% HIRI model, pretreated them with L-NAT or saline vehicles. Morphologic changes of hepatocytes were evaluated by HE staining. The hepatocytes of I/R group showed obvious characteristics of tissue damage, such as edema, ballooning hydropic degeneration, membrane rupture and neutrophil infiltration (Fig. 1A). And the Suzuki score was significantly higher than that in the sham group. L-NAT pretreatment effectively inhibited the I/R-induced changes of hepatocytes (Fig. 1B), indicating that L-NAT could alleviate the morphological changes of liver cells caused by ischemia-reperfusion.

As shown in Fig. 1C, the level of serum ALT in the I/R group was significantly higher than that in the sham group (*p* < 0.01), manifesting damage of hepatocytes in the I/R group. Compared to the I/R group, the level of serum ALT was significantly lower than that of the I/R + L-NAT group (*p* < 0.01), which indicated that L-NAT could prevent liver injury caused by HIRI.

**Figure 2:**
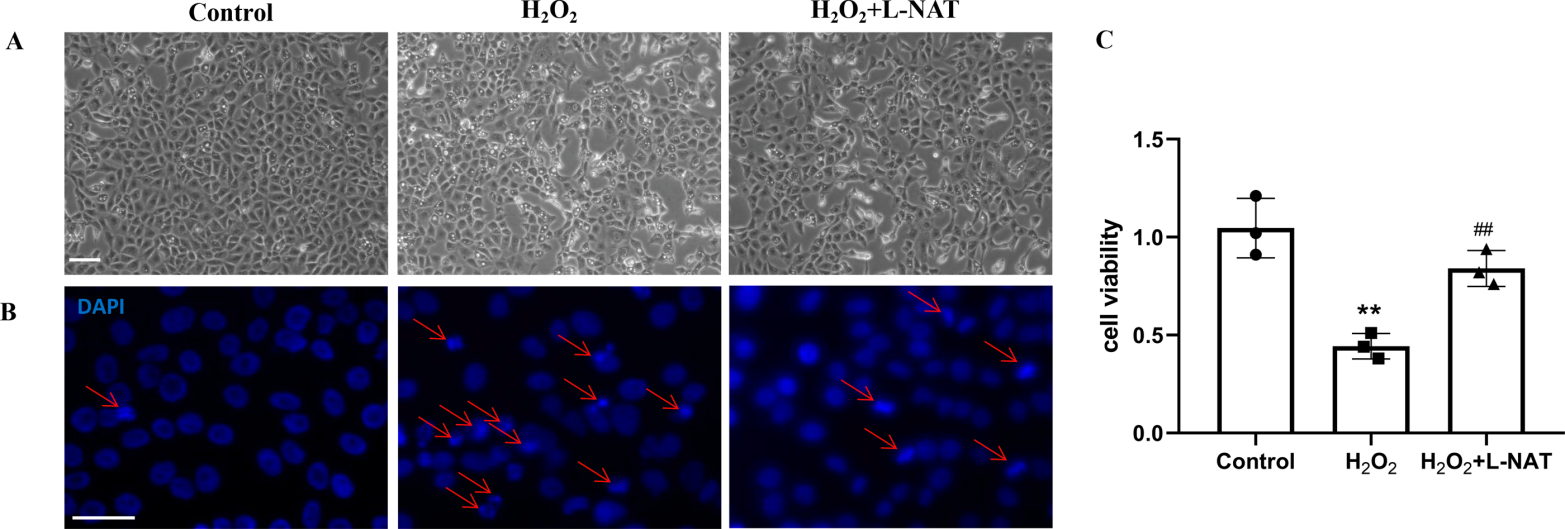
H_2_O_2_ induced cell death in BRL cells, while L-NAT protected them from H_2_O_2_-mediated cell death. The morphologic changes of BRL cells showed under inverted phase-contrast microscope (A). Compared to the control group, BRL cells of H_2_O_2_-induced group became scarcer. Part of them shrinkage with a round and bright nucleus. The H_2_O_2_-mediated cell death was further studied by DAPI staining under fluorescence microscopy (B). Cell viability was determined by CCK-8 colorimetric kit (C). Scale bar: 50 µm.

As described in our recent reports, the H_2_O_2_-induced oxidative damaged model was established with co-incubation of 200 µM H_2_O_2_ and BRL cells for 18 h (Wang et al., 2019). To evaluate the cytoprotective effect of L-NAT on hepatocytes *in vitro*, we next investigated the morphological changes of BRL cells using the H_2_O_2_-induced oxidative damaged model with or without L-NAT. As shown in Fig. 2A, compared to the control group, BRL cells of H_2_O_2_-induced group became scarcer. Part of them shrunk with a round and bright nucleus. In agreement with the results under phase contrast microscopy, DAPI staining also confirmed the change of nucleus induced by H_2_O_2_ (Fig. 2B). However, all those morphological changes of BRL cells were reversed by L-NAT pretreated. And L-NAT administration could improve the decline of cell viability which was induced by H_2_O_2_ (Fig. 2C), which implied that L-NAT could reduce H_2_O_2_-induced oxidative stress damage in BRL cells.

### Effects of L-NAT on NLRP3 and ASC after HIRI

**Figure 3:**
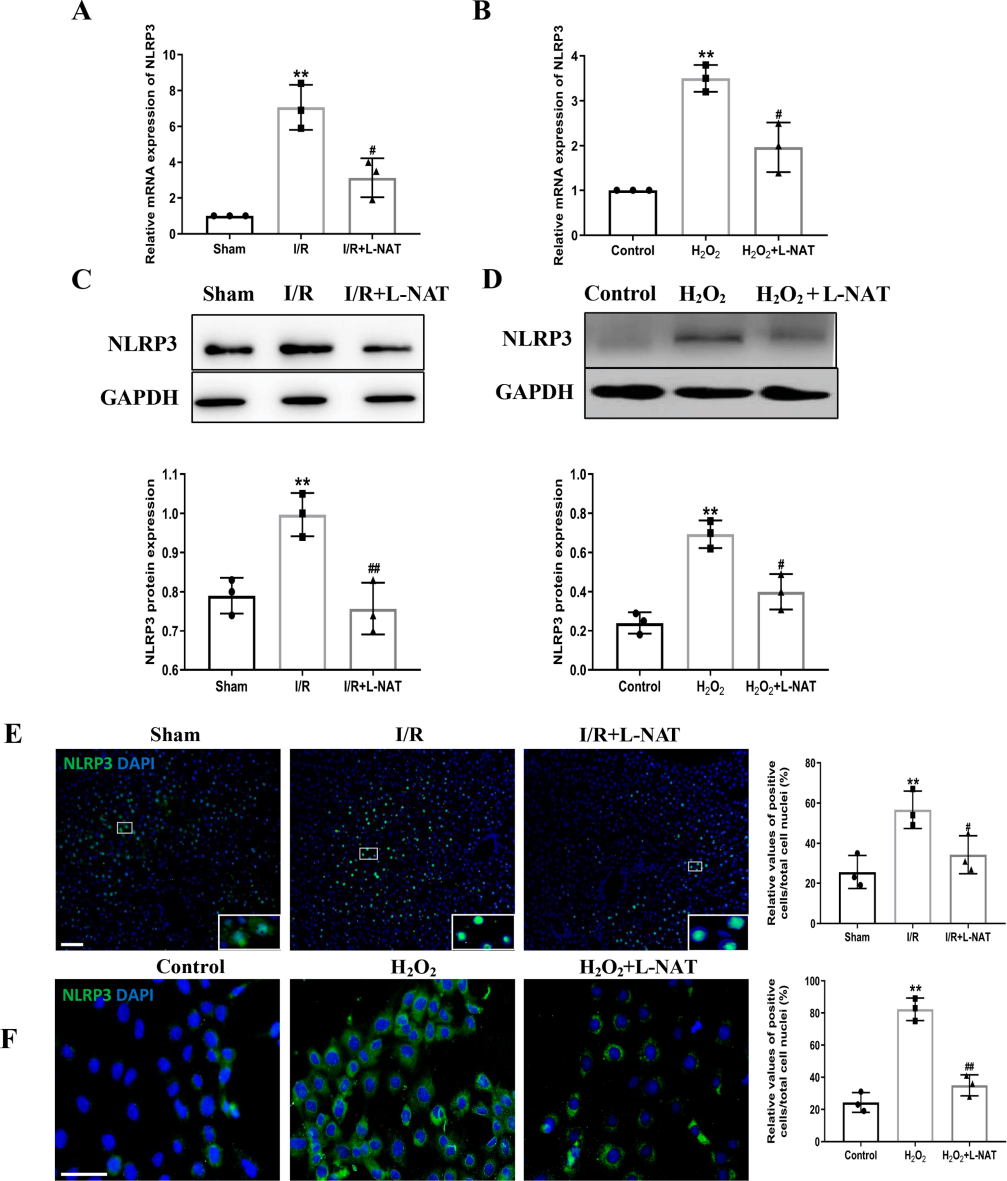
The expression of NLRP3 had a significant up-regulation in HIRI and L-NAT administration rescued the changes of NLRP3. The L-NAT (10 mg/kg) or saline was administered by IP injection 30 min before ischemia (A, C, E). The BRL cells were treated with 200 µM H_2_O_2_ for 18 h with or without L-NAT (B, D, F). Subsequently, the effect of L-NAT on the expression of NLRP3 was detected by qRT-PCR (A, B), WB (C, D) and immunofluorescence staining (E, F). The data were the mean ± SD (One-way ANOVA, *n* = 3), ^∗∗^*p* < 0.01 compared with the related sham or control group, ^##^*p* < 0.01, ^#^*p* < 0.05 compared with the related I/R or H_2_O_2_ group. Scale bar: 50 µm.

**Figure 4:**
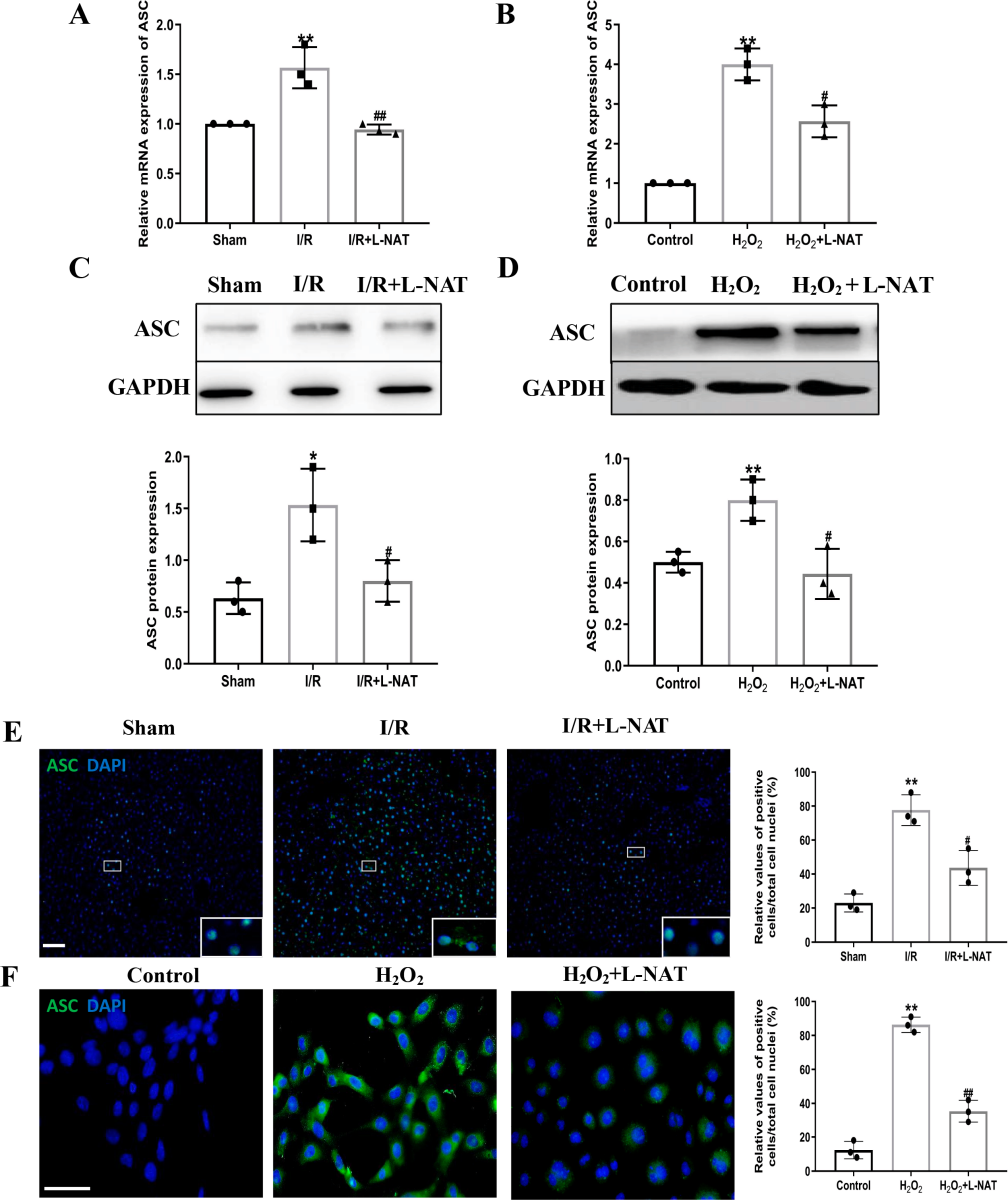
L-NAT inhibited the expression of ASC during HIRI. The effects of L-NAT on the expression of ASC were detected by qRT-PCR (A, B), WB (C, D) and immunofluorescence staining (E, F). The data were the mean ± SD (One-way ANOVA, *n* = 3), ^∗∗^*p* < 0.01, ^∗^*p* < 0.05 compared with the related sham or control group, ^##^*p* < 0.01, ^#^*p* < 0.05 compared with the related I/R or H_2_O_2_ group. Scale bar: 50 µm.

NLRP3 inflammasome mediates the ischemia-reperfusion injury in organs such as liver, kidney, brain and heart. ASC, an important adaptor protein in NLRP3 inflammasome, recruits pro-Caspase-1 into the complex through its CARD domain, providing a molecular platform for the activation of Caspase-1 (Man & Kanneganti, 2015). Therefore, the expressions of NLRP3 and ASC of hepatocytes were investigated during the process of HIRI models with or without L-NAT both *in vitro* and *in vivo*, which were made by BRL cells and SD rats, respectively. In vivo, results of qRT-PCR showed that the expressions levels of NLRP3 and ASC mRNA were higher in the I/R group than that in the sham group, and the L-NAT pre-treatment significantly rescued these changes (*p* < 0.01, Figs. 3A, 4A). Results of Western blotting also showed an increase than those in the sham group (Figs. 3C, 4C). Immunofluorescence staining showed that NLRP3 and ASC positive hepatocytes were consistent with Western blotting results (Figs. 3E, 4E), indicating that L-NAT significantly inhibited the increased expressions of NLRP3 and ASC after HIRI. Subsequently, we further examined the expressions of NLRP3 and ASC in H_2_O_2_-triggered BRL cells injury model.

Similar to the *in vivo* results, the expressions of NLRP3 and ASC elevated by H_2_O_2_ insult, and L-NAT effectively antagonized H_2_O_2_-induced up-regulation of them (Figs. 3B, 3D, 3F, 4B, 4D, 4F). Taken together, these results provided strong evidence that L-NAT could inhibit the expressions of NLRP3 and ASC during HIRI.

### Effects of L-NAT on Caspase-1 after HIRI

**Figure 5:**
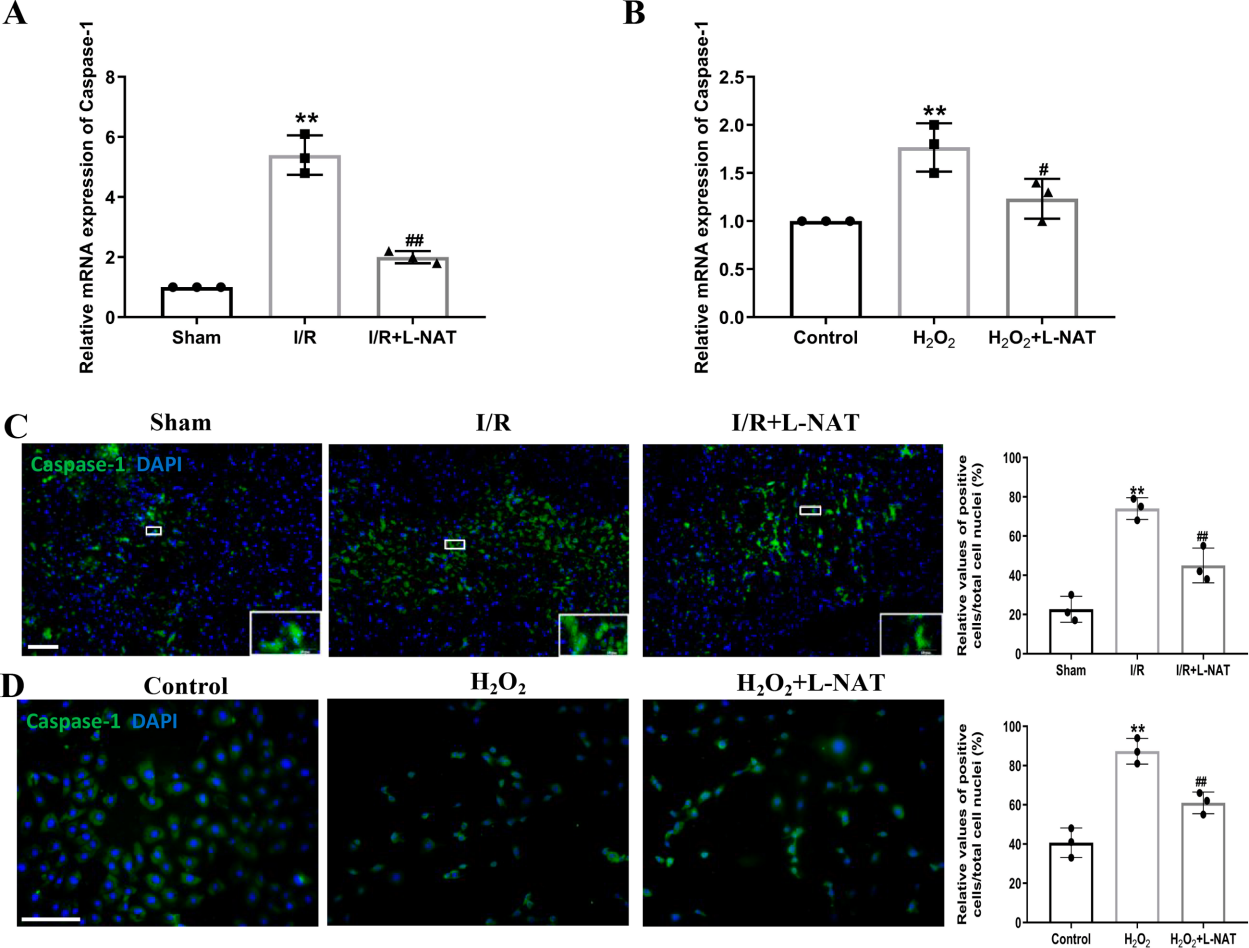
L-NAT activated the Caspase-1 during HIRI. The hepatic tissues and the BRL cells were extracted and analyzed by qRT-PCR (A, B) or immunofluorescent stained with cleaved-Caspase-1 antibody (C, D). The data were the mean ± SD (One-way ANOVA, *n* = 3), ^∗∗^*p* < 0.01 compared with the related sham or control group, ^##^*p* < 0.01, ^#^*p* < 0.05 compared with the related I/R or H_2_O_2_ group. Scale bar: 50 µm.

The occurrence of pyroptosis depends on Caspase-1, which is a key protein in the pyroptosis pathway of cells (Gu et al., 2021). Previous studies have shown that activated Caspase-1 could mediate inflammation and pyroptosis by activating IL-1β and Recombinant Gasdermin D (GSDMD), respectively (Schneider et al., 2017). Therefore, we next investigated the expression of Caspase-1 by qRT-PCR and immunofluorescence staining. The mRNA expression of Caspase-1 in the I/R group revealed an increase than that in the sham group (*p* < 0.01), and L-NAT administration could reverse those changes (*p* < 0.01, Fig. 5A). Immunofluorescence staining also showed a significant increase of the activated Caspase-1 (Fig. 5C). In vitro, the mRNA level of Caspase-1 also showed a dramatic increase in the H_2_O_2_-treated group compared with the control group (*p* < 0.01). In parallel, compared to the H_2_O_2_ group, L-NAT intervention significantly reduced the mRNA expression of Caspase-1 compared with the H_2_O_2_ group (*p* < 0.05, Fig. 5B). The change of immunopositive reaction of Caspase-1 was similar to that of qRT-PCR (Fig. 5D). The above results indicated that L-NAT pretreatment could reduce the activation of Caspase-1 after HIRI.

### Effects of L-NAT on the expression and secretion of IL-1 in BRL cells after H_2_O_2_- induced

Interleukin-1β (IL-1β), an important mediator of inflammatory response after ischemia-reperfusion, could induce the synthesis of other inflammatory factors, chemokines and adhesion molecules, etc. The cascade magnification reaction, which causes pathological inflammatory response, is an important “executor” of cell pyroptosis-induced cell inflammatory death (Liang et al., 2021).

**Figure 6:**
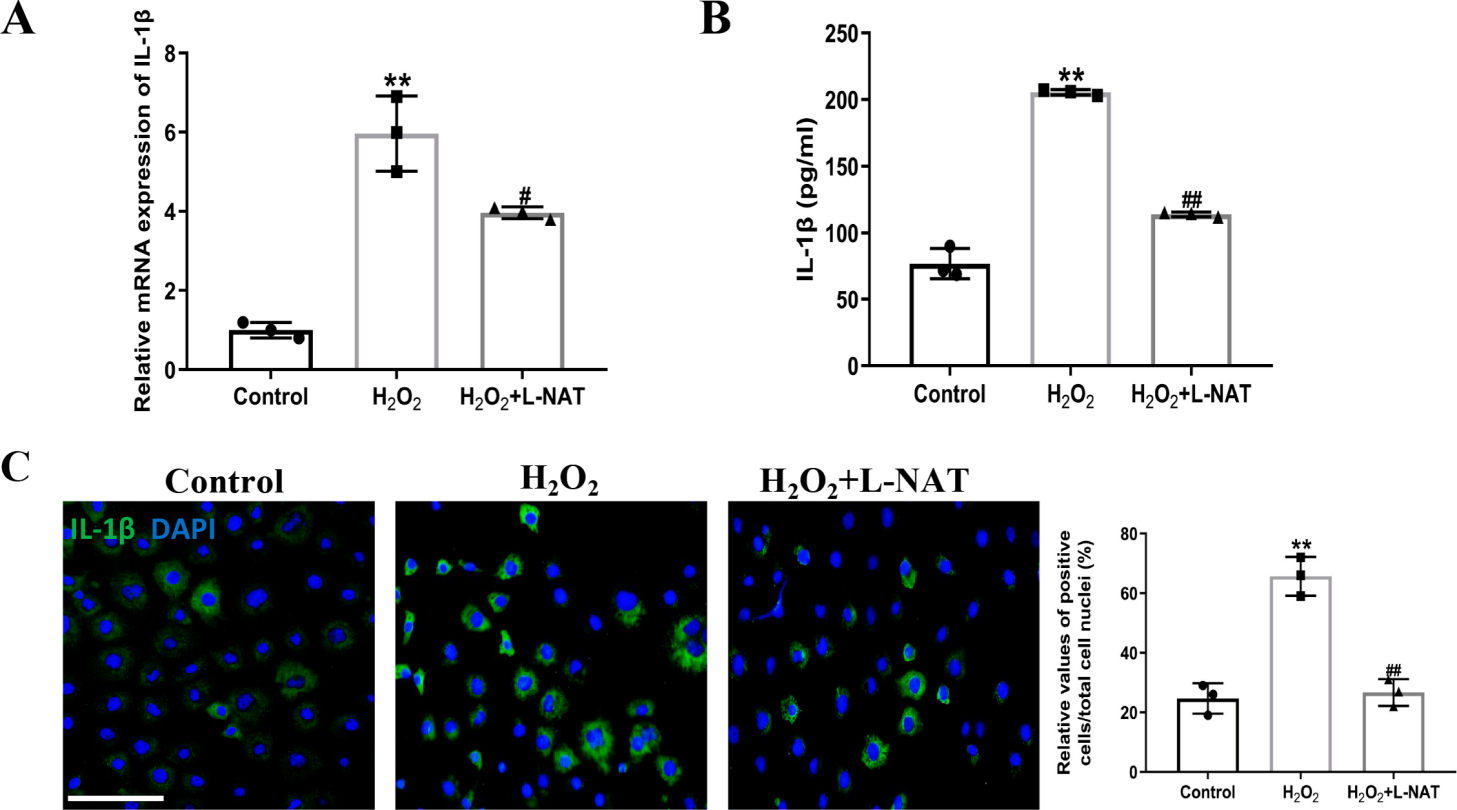
L-NAT ameliorated the expression and secretion of IL-1β. The BRL cells were treated 200 µM H_2_O_2_ for 18 h with or without L-NAT. The whole-cell lysates were extracted and analyzed by qRT-PCR (A). The supernatant was collected to analyze the secretion of IL-1β (B). The expression of IL-1β was also showed by immunofluorescence staining (C). Results showed that the expression and secretion of IL-1β were increased in the group and decreased by L-NAT administration. The data were the mean ± SD (One-way ANOVA, *n* = 3), ^∗∗^*p* < 0.01 compared with the control group, ^##^*p* < 0.01, ^#^*p* < 0.05 compared with the H_2_O_2_ group. Scale bar: 50 µm.

The effect of L-NAT on the expression and secretion of IL-1β in BRL cells after H_2_O_2_ induction was detected by qRT-PCR, ELISA and immunofluorescence staining. The qRT-PCR analysis showed a significant expression level of IL-1β in the H_2_O_2_ group comparison with the control group (*p* < 0.01). In contrast, treatment with L-NAT (10 µM) significantly reduced the expression of IL-1β compared with the H_2_O_2_ group (*p* < 0.05, Fig. 6A). As shown in Fig. 6B, compared with H_2_O_2_ group, L-NAT administration markedly decreased the secretion of IL-1β (*p* < 0.01). Immunofluorescence staining confirmed the changes of IL-1β in oxidative stress injury model and the protective effect of L-NAT (Fig. 6C). Taken together, these results provided strong evidence that L-NAT pretreatment could inhibit the expression and secretion of IL-1β during HIRI.

### Effects of L-NAT on TLR4 and NF-B after HIRI

**Figure 7:**
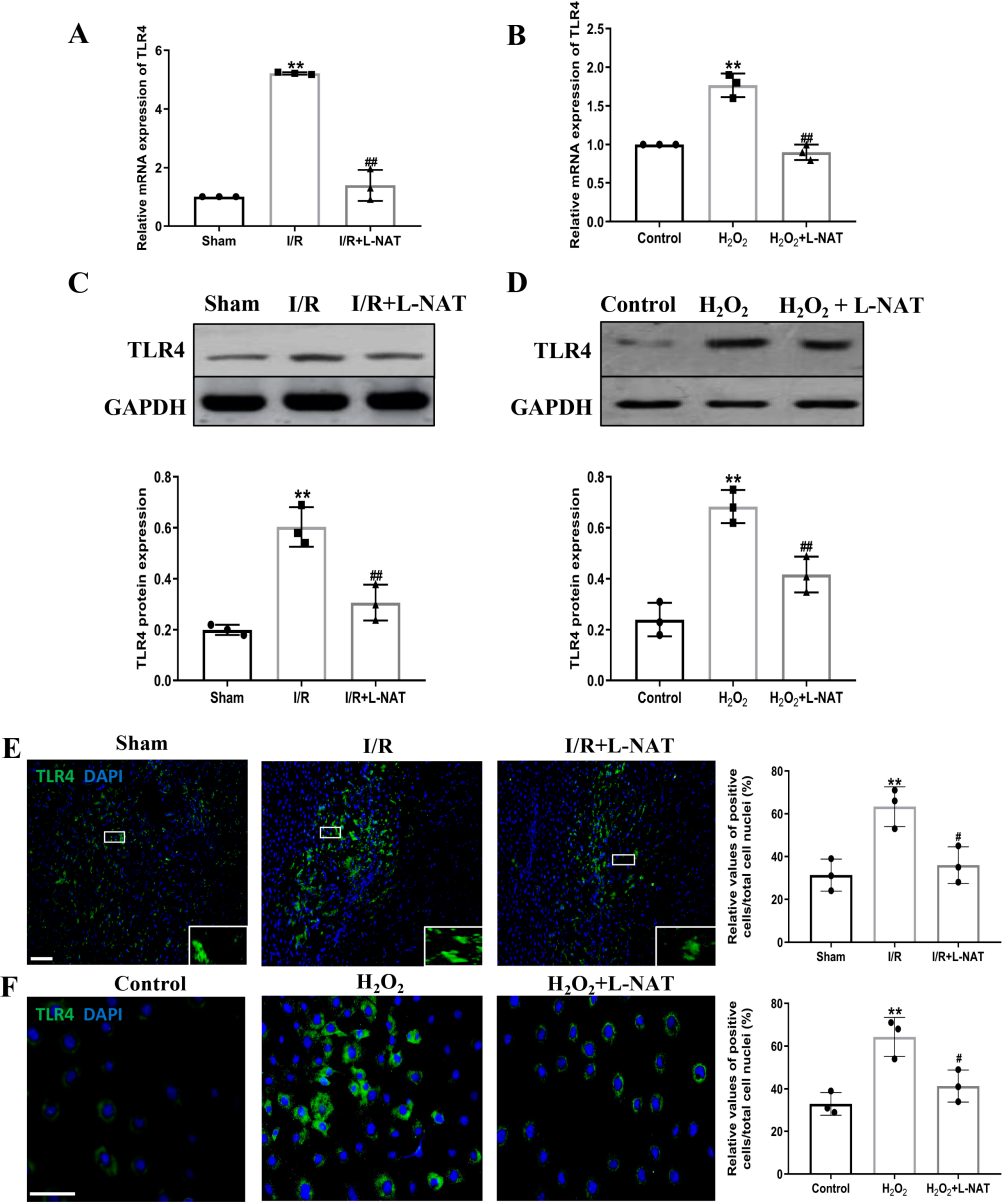
L-NAT reversed the up-regulated TLR4 induced by HIRI. The effects of L-NAT on the expression of TLR4 were detected by qRT-PCR (A, B), WB (C, D) and immunofluorescence staining (E, F). Results showed that the expression of TLR4 was up-regulated by HIRI *in vivo* (A, C, E) and *in vitro* (B, D, F). Incubation with L-NAT significantly reduced the expression of TLR4. The TLR4 expression was also delectated by immunofluorescence staining. A high level of TLR4 immunoreactivity was observed in the I/R or H_2_O_2_ groups compared to the sham or control groups and those changes were partially revised by L-NAT administration. The data were the mean ± SD (One-way ANOVA, *n* = 3), ^∗∗^*p* < 0.01 compared with the related sham or control group, ^##^*p* < 0.01 compared with the related I/R or H_2_O_2_ group. Scale bar: 50 µm.

**Figure 8:**
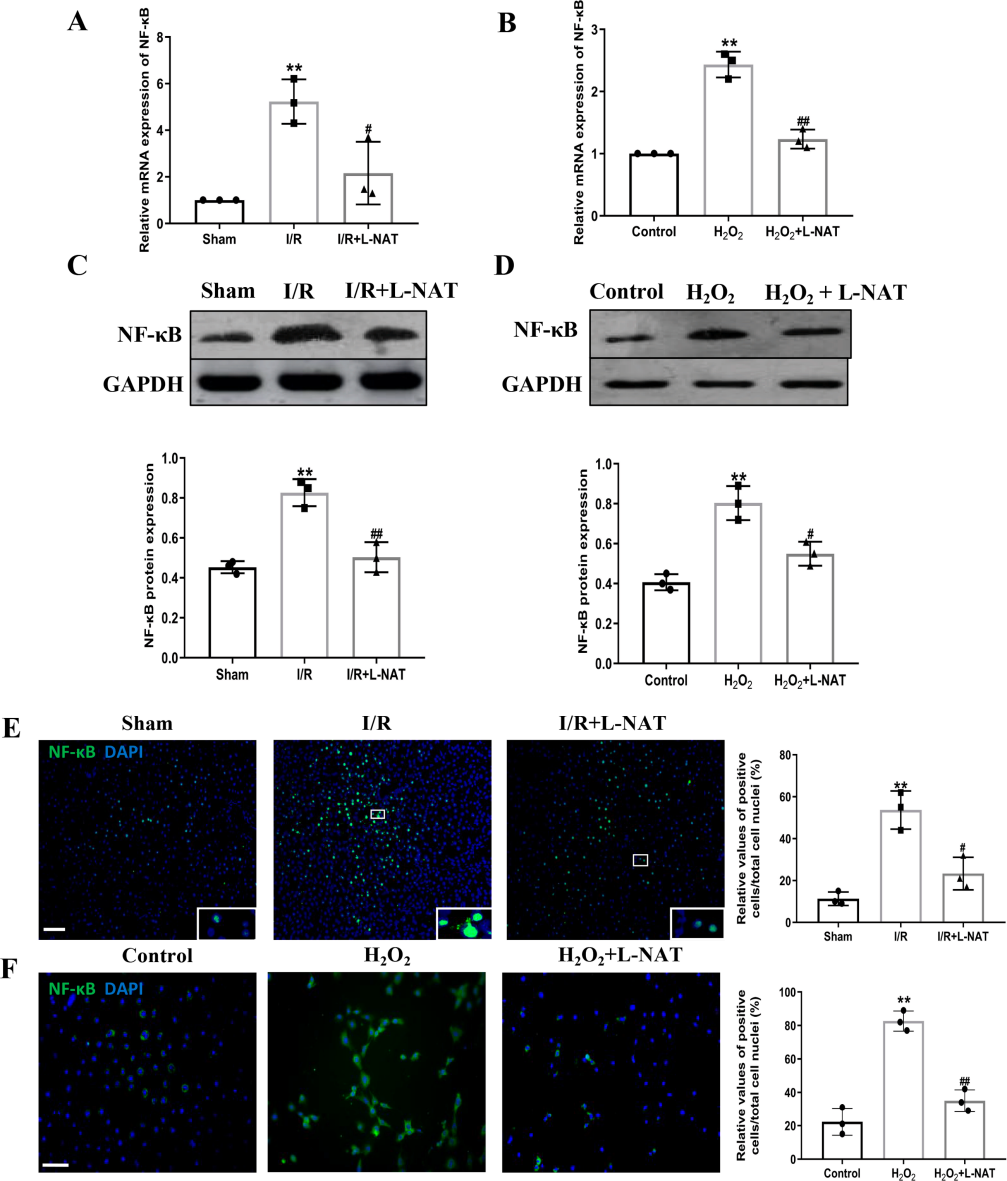
Induction of NF-κB in hepatocytes following HIRI was rescued by L-NAT administration. The hepatic tissues and the BRL cells were analyzed by qRT-PCR (A, B) and WB (C, D). The expression of NF-κB had a significant up-regulation in HIRI and L-NAT administration attenuated the expression of NF-κB (A, C). The expression of NF-κB was also showed by immunofluorescence staining (E, F), which consistent with qRT-PCR results. The data were the mean ± SD (One-way ANOVA, *n* = 3), ^∗∗^*p* < 0.01 compared with the related sham or control group, ^##^*p* < 0.01, ^#^*p* < 0.05 compared with the related I/R or κ group. Scale bar: 50 µm.

TLR4 was a critical factor in the inflammatory process, which could up-regulate the expression of NF-κB and induce its nuclear translocation. The TLR4/NF-κB signaling pathway could promote the production of inflammatory cytokines. Subsequently, the expressions of TLR4 and NF-κB evaluated by qRT-PCR, Western blotting and Immunofluorescence staining. The mRNA level of TLR4 and NF-κB in the I/R group revealed an increase than that in the sham group (*p* < 0.01), and L-NAT administration could reverse those changes (*p* < 0.05, Figs. 7A, 8A). The results of Western blotting also showed an increase than those in the sham group (*p* < 0.01, Figs. 7C, 8C). Immunofluorescence staining showed that TLR4 and NF-κB positive reaction in hepatocytes was consistent with Western blotting results (Figs. 7E, 8E), indicating that L-NAT significantly inhibited the increase of TLR4 and NF-κB after HIRI. Subsequently, we further examined the expressions of TLR4 and NF-κB in H_2_O_2_-triggered BRL cells injury model.

Similarly, compared to the H_2_O_2_ group, a significant reduction in TLR4 and NF-κB expression levels was noted following treatment with L-NAT (*p* < 0.01, Figs. 7B, 7D, 7F, 8D, 8F), suggesting that L-NAT pretreatment could reduce the expressions of TLR4 and NF-κB in oxidative stress injury of liver cells.

## Discussion

To study the protective effect of L-NAT on NLRP3 inflammasome and related inflammatory cytokines, we utilized rat HIRI model and H_2_O_2_-induced BRL cell oxidative stress damage model. The results indicated that some inflammatory cytokines, such as TLR4, NF-κB, and IL-1β, were passively produced during the process of HIRI. While L-NAT pretreatment could inhibit the expressions of inflammatory factors suggesting that it could inhibit the inflammatory response induced by ischemia-reperfusion. In addition, we also discovered that L-NAT could inhibit the expressions of NLRP3, ASC, Caspase-1 and the NLRP3 inflammasome related protein, which verified L-NAT could block the activation of NLRP3 inflammasome. All these results demonstrated that L-NAT could attenuate HIRI by regulating TLR4/NLRP3 signal, which would provide experimental data for the application of L-NAT in the field of liver diseases.

HIRI is a major complication that affects the efficacy and mortality of liver surgery. Although a large number of basic and clinical studies have been carried out, the mechanism of ischemia-reperfusion injury, which may be related to oxidative stress injury, calcium overload and inflammation, has not yet been fully clarified. At present, more and more researches on the mechanism of HIRI have focused on the inflammatory response. Recently, as the core part of the inflammatory response, the role of inflammasomes in HIRI has attracted much attention. Inflammasomes are multi-protein complexes formed by assembled intracytoplasmic pattern recognition receptors (PRRs). By now, four inflammasomes, including NLRP1, NLRP3, NLRC4 and AIM2, have been confirmed. Among them, NLRP3 is the most important inflammasome, and is regarded as a sensor and effector of HIRI (Zhai et al., 2013; Szabo & Petrasek, 2015). The NLRP3 inflammasomes are composed of receptor protein NLRP3, apoptosis-related dot-like protein ASC and effector protein pro-Caspase-1. Once NLRP3 inflammasome is activated, the inactive pro-Caspase-1 was cleaved to form cleaved Caspase-1, and then cleaved pro-IL-1β into mature IL-1β. Activated IL-1β induces the synthesis of inflammatory factors and chemokines. Therefore, IL-1β, an important inflammatory signaling amplification factor, plays a protective role on HIRI (Furuichi et al., 2006; Ouzounidis et al., 2016). Using a small hairpin RNA (shRNA) plasmid targeting NALP3/NLRP3, Zhu et al. (2011) investigated the function and potential mechanisms of NALP3 in a murine model of HIRI. They confirmed the protective effect of NALP3 silencing in HIRI, which is associated with reductions of IL-1β, IL-18, TNF-α, IL-6, and HMGB1 through down-regulating the activation of Caspase-1 and NF-κB. Inoue et al. (2014) found that NLRP3^−∕−^ mice had significantly less liver injury, inflammatory responses, reactive oxygen species production and apoptosis during the process of HIRI compared to the wild-type mice. Kamo et al. (2013) showed that the ASC/Caspase-1/IL-1β signaling mediates the inflammatory response by triggering the induction of HMGB1 during HIRI. Subsequently, ASC deficiency and neutralization of IL-1β ameliorated the hepatocellular apoptosis and caused down-regulation of HMGB1-mediated, TLR4-driven inflammation. Glycyrrhizic acid, docosahexaenoic acid (DHA), linoleic acid, Chinese medicine Xuebijing and T3 had been confirmed to reduce HIRI by inhibiting the activation of NLRP3 inflammasome (Liu et al., 2015; Vargas & Videla, 2017; Li et al., 2018). Taken together, the above results suggested that NLRP3 inflammasome may be involved in the occurrence of HIRI. As described in our recent reports, L-NAT could alleviate HIRI, but whether L-NAT-mediated protection is related to NLRP3 inflammasome remains unknown (Wang et al., 2019). The present study demonstrated that HIRI markedly increased the expression of NLRP3, ASC, and Caspase-1 *in vivo* and *in vitro*, which are consistent with the results of Xu et al. (2017). The above researches suggested that the NLRP3 inflammasome serves an important role in the occurrence and development of HIRI.

The DAMPs released after ischemia-reperfusion could directly activate the NLRP3 inflammasome. On the one hand, DAMPs promote the activation of Caspase-1 by cutting pro-IL-1β and pro-IL-18 to form mature IL-1β and IL-18. On the other hand, it can also be recognized by TLR4, which is rapidly activated after binding with ligand to activate NF-κB, a transcriptional regulator that mediates the expression of a variety of genes and participates in signal transduction and amplification cascades during inflammation (Man & Kanneganti, 2015; Lu & Wu, 2015). Previous studies have demonstrated that TLR4 plays a crucial role in the pathogenesis of HIRI (Youn et al., 2005). TLR4 mutant mice have less liver function and morphological damage as compared with wild-type mice (Wang et al., 2007). The levels of transaminase and the expression of TNF-α in mice lacking TLR4 were drastically lower after ischemia-reperfusion (Wu et al., 2004). Consistent with the total form of protein expression trend we observed, myocardial I/R injury in rats leads to overtly elevated expression levels of p-TLR4 and p-NF-κB, and decreased expression of miR-21 (Pan et al., 2018). Furthermore, some drugs, such as dendrobium officinale polysaccharides, could also display remarkable anti-inflammation and hepatoprotective effects via the TLR4/NF-κB signaling pathway (Yang et al., 2020), which indicated that ischemia-reperfusion injury is related to the activation of the TLR4/NF-κB signaling pathway.

A large body of evidence indicates that TLR4 could also mediate the activation of NLRP3 inflammasomes. The activation of NLRP3 inflammasome requires the cooperation of two signals, pre-excitation signal and activation signal. The pre-excitation signal binds to ligands through toll-like receptors, activating NF-κB and up-regulating the transcription of NLRP3 and pro-IL-1β. The activation signal promotes the assembly and activation of NLRP3 inflammasomes by a variety of stimulating factors to increase the release of mature IL-1β. It shows that the activation of the TLR4/NF-κB signaling pathway can only generate inactive pro-IL-1β, while the formation and activation of NLRP3 inflammasome can mediate the cleavage and maturity of pro-IL-1β. TLR4 and NLRP3 inflammasome cooperate in the production and secretion of IL-1β. In addition, TLR4 can promote the deubiquitination of NLRP3, which provides materials for the assembly of NLRP3 inflammasomes (Juliana et al., 2012). In conclusion, NLRP3 inflammasome and TLR4/NF-κB signal cooperated to regulate the inflammatory response induced by ischemia-reperfusion. In the present study, we showed that the expressions of TLR4 and NF-κB were elevated during HIRI both *in vitro* and *in vivo*. Meanwhile, we also showed a markedly increased expression of IL-1β and other inflammatory cytokines induced by HIRI. These data verified the role of TLR4/NF-κB signal-mediated inflammatory reaction in HIRI. Therefore, inhibiting the expression of TLR4 or blocking the downstream signaling pathway might be a potential strategy of ischemia-reperfusion injury treatment.

The wide expression of NK-1R in various organs and the role in many diseases make the highly selective agonists and antagonists of NK-1 receptor become a research hotspot. Growing evidence indicated that NK-1R involved in the occurrence and development of the inflammation-related disease including pneumonia (Weisshaar & Winkelstein, 2014), rheumatoid arthritis (Makino et al., 2012), HIV/SIV encephalitis (Vinet-Oliphant et al., 2010), sepsis (Etogo et al., 2008), asthma (Boot et al., 2007) and skin itching (Costa et al., 2006), etc. These data implied that NK-1R antagonists may be used as therapeutic marker for inflammation-related disease. As one kind of NK-1R antagonists, L-NAT could not only inhibit the binding of SP and NK-1R, but also block the excretion of Cyt C from mitochondria. Used the calcium ion-induced mitochondrial Cyt C release model, Li et al. (2015) confirmed that L-NAT could inhibit the release of Cyt C from mitochondria. Subsequently, Li et al. (2015) demonstrated that L-NAT could delay the onset, decline the death of mSOD1G93A ALS mice and decrease the H_2_O_2_ and OGD-induced NSC-34 cell damage and ST14A striatal cell death induced by mutant huntingtin protein (Li et al., 2015). Other researchers have successively confirmed the protective effect of L-NAT on PD (Thornton et al., 2014), Alzheimer’s disease (Fernandes et al., 2018), traumatic brain injury (Jha, Kochanek & Simard, 2019; Donkin et al., 2009), stroke (Turner & Vink, 2012), opioid withdrawal (Tumati et al., 2012) and OTA-induced HEK-293 cells (Agarwal et al., 2020) damage. All these results indicated that L-NAT may reduce cell death by inhibiting oxidative stress and protein synthesis, stabilizing mitochondrial membrane potential, and reducing mitochondrial dysfunction. Li et al. (2015) demonstrated that L-NAT delayed the disease onset of ALS, extended survival, reduced the release of cytochrome c/smac/AIF, increased B-cell lymphoma-2 (Bcl-2) levels, inhibited the activation of Caspase-3, ameliorated motor neuron loss, suppressed inflammation and restored NK-1R levels, which suggested that L-NAT could provide protection in ALS by inhibiting mitochondrial cell death pathways, inflammation and NK-1R down regulation. Using the H_2_O_2_ induced oxidative damaged model of BRL cells, we previously confirmed that exogenous SP induced the death of BRL cells in a dose-dependent manner (Xiao et al., 2016). H_2_O_2_ increases the release of SP in a time-dependent manner. L-NAT could inhibit the release of mitochondrial pro-apoptotic factors and the activation of Caspase cascade, confirming that L-NAT had a neuroprotective effect in the ALS cell model. Previous results of our laboratory showed that SP aggravated the oxidative damage of NSC-34 cell and L-NAT could not only prevent the secretion of SP, but also inhibit the activation of Caspase-1 and IL-1β, indicating that L-NAT may be the effective agent in the treatment of ALS (Sirianni et al., 2015). Used partial HIRI animal models and H_2_O_2_-induced BRL cell oxidative injury models, we not only observed the protective effect of L-NAT on HIRI (Wang et al., 2019); (Li et al., 2020a), but also demonstrated its protection on the striatum and intestinal injury induced by HIRI (Wang et al., 2017; Zhao et al., 2016). At present, the molecular mechanisms underlying the hepatic-protection of L-NAT remains unknown. We studied the change of NLRP3 inflammasome during the process of HIRI in rats, by measurement of the expression of NLRP3, ASC, Caspase-1, IL-1 β, TLR4 and NF-κB. We found that administration of L-NAT effectively inhibited the activation of NLRP3 inflammasome, prevented the expressions of TLR4, NF-κB and IL-1 *β*, and the cleavage of Caspase-1, and improved the morphological and functional changes of hepatocytes induced by HIRI. Based on the above results, we speculated that the hepatic-protective effect of L-NAT was achieved by regulating TLR4/NLRP3 signal.

## Conclusion

The present study evaluated the effects of L-NAT on the hepatocytes death, the activation of NLRP3 inflammasome and the expression of inflammatory cytokines, suggested that L-NAT alleviated HIRI in rat via downregulating the protein expression of NLRP3, which was related to the TLR4/NF-κB signaling pathway.

##  Supplemental Information

10.7717/peerj.11909/supp-1 Supplemental Information 1 Raw data L-NAT alleviates hepatic ischemia-reperfusion injury.

10.7717/peerj.11909/supp-2 Supplemental Information 2 Western Bolt

10.7717/peerj.11909/supp-3 Supplemental Information 3 Author Checklist

## Additional Information and Declarations

### Data Availability

The following information was supplied regarding data availability: The raw measurements are available in the Supplemental Files.
