# Supplementary figures and images for: N-acetyl-L-tryptophan attenuates hepatic ischemia-reperfusion injury via regulating TLR4/NLRP3 signaling pathway in rats
Source: PeerJ. 2021 Aug 10;9:e11909. doi: 10.7717/peerj.11909 (PMC8362669; doi:10.7717/peerj.11909)

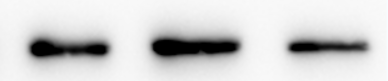

Supplement: Supplemental Information 2 [file peerj-09-11909-s002.zip › Western Bolt/Fig3/Fig3C NLRP3.png]

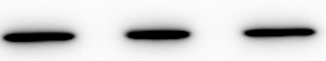

Supplement: Supplemental Information 2 [file peerj-09-11909-s002.zip › Western Bolt/Fig3/Fig3C-GAPDH.png]

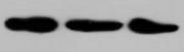

Supplement: Supplemental Information 2 [file peerj-09-11909-s002.zip › Western Bolt/Fig3/Fig3D-GAPDH.png]

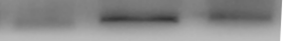

Supplement: Supplemental Information 2 [file peerj-09-11909-s002.zip › Western Bolt/Fig3/Fig3D-NLRP3.png]

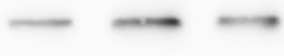

Supplement: Supplemental Information 2 [file peerj-09-11909-s002.zip › Western Bolt/Fig4/Fig4C-ASC.png]

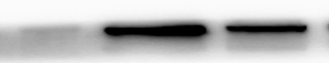

Supplement: Supplemental Information 2 [file peerj-09-11909-s002.zip › Western Bolt/Fig4/Fig4D-ASC.png]

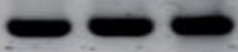

Supplement: Supplemental Information 2 [file peerj-09-11909-s002.zip › Western Bolt/Fig7/Fig7C-GAPDH.png]

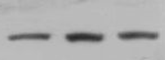

Supplement: Supplemental Information 2 [file peerj-09-11909-s002.zip › Western Bolt/Fig7/Fig7C-TLR4.png]

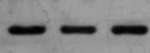

Supplement: Supplemental Information 2 [file peerj-09-11909-s002.zip › Western Bolt/Fig7/Fig7D-GAPDH.png]

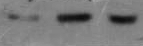

Supplement: Supplemental Information 2 [file peerj-09-11909-s002.zip › Western Bolt/Fig7/Fig7D-TLR4.png]

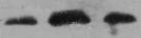

Supplement: Supplemental Information 2 [file peerj-09-11909-s002.zip › Western Bolt/Fig8/Fig8C-NF-╬║B.png]

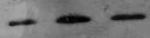

Supplement: Supplemental Information 2 [file peerj-09-11909-s002.zip › Western Bolt/Fig8/Fig8D-NF-╬║B.png]

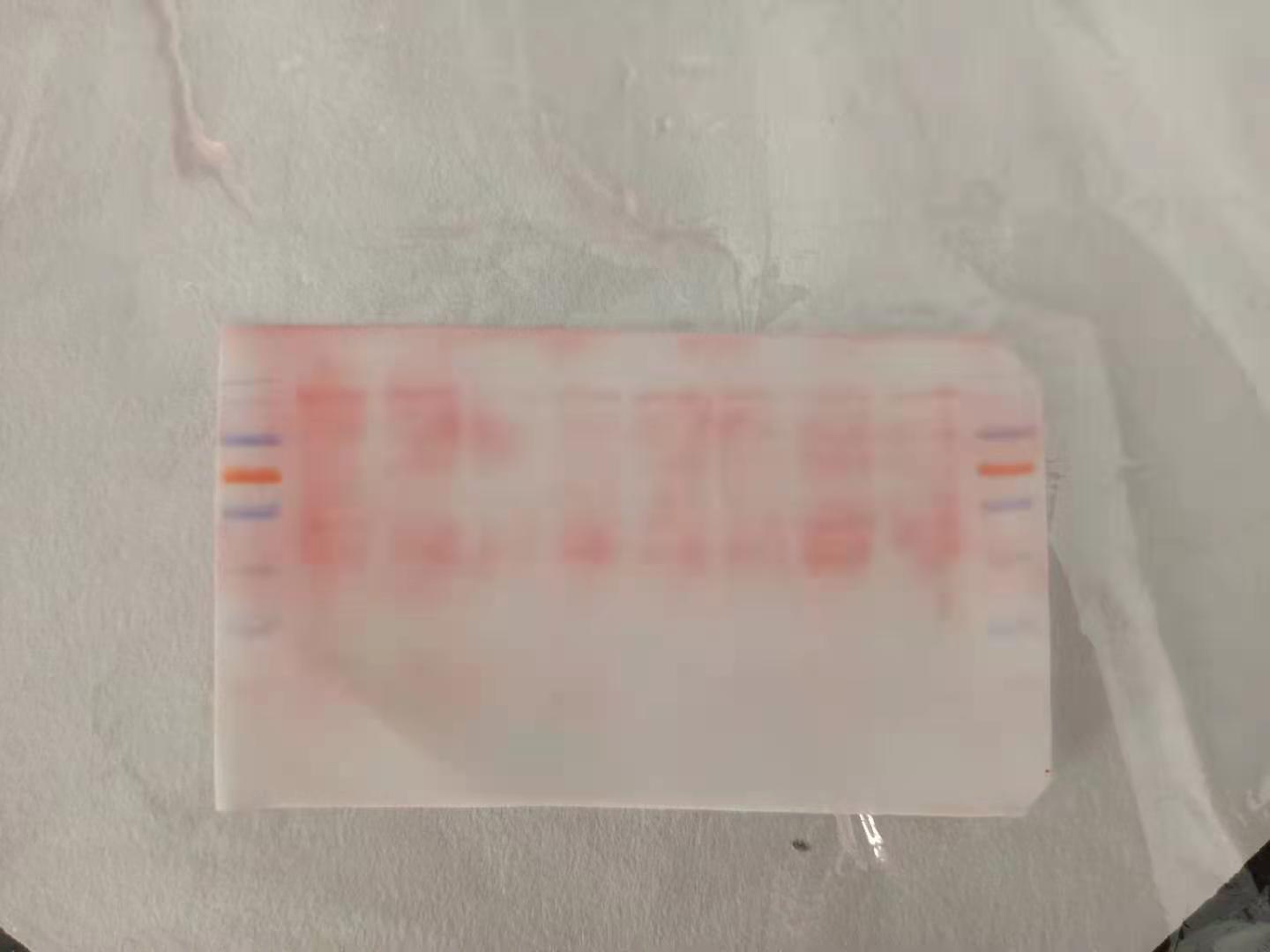

Supplement: Supplemental Information 2 [file peerj-09-11909-s002.zip › Western Bolt/WB1.jpg]

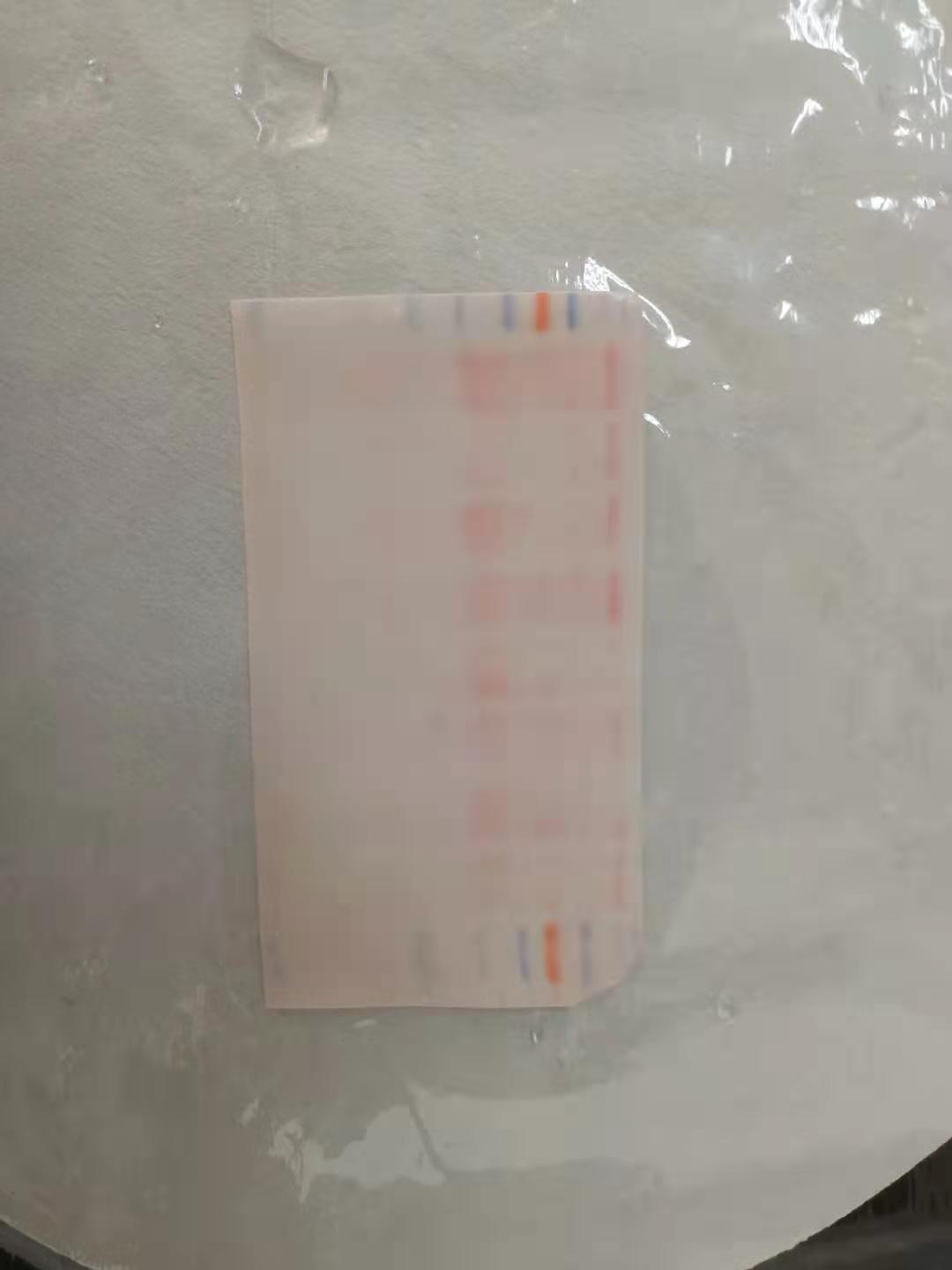

Supplement: Supplemental Information 2 [file peerj-09-11909-s002.zip › Western Bolt/WB2.jpg]

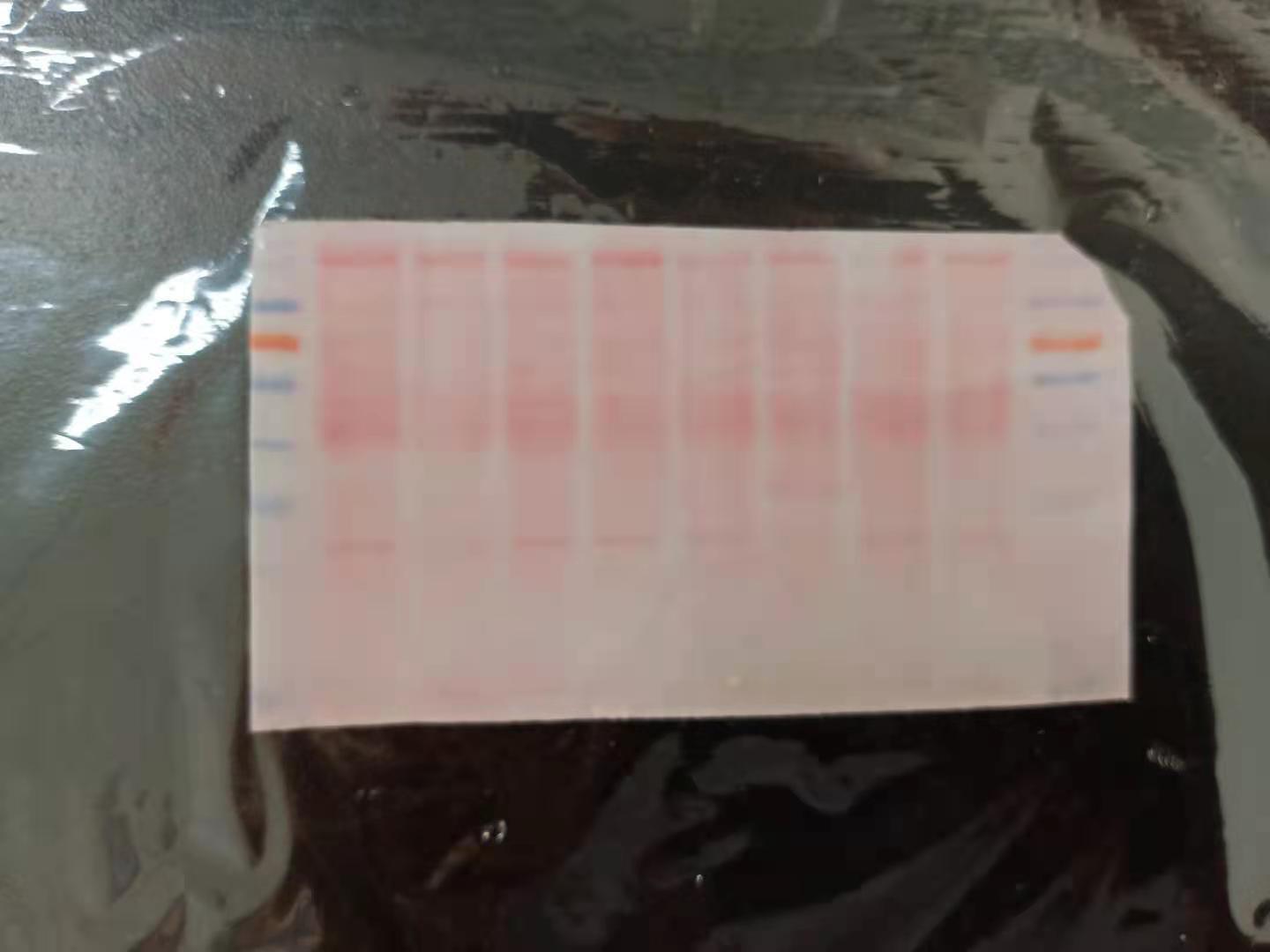

Supplement: Supplemental Information 2 [file peerj-09-11909-s002.zip › Western Bolt/WB3.jpg]
